# Differential Neural Processing during Motor Imagery of Daily Activities in Chronic Low Back Pain Patients

**DOI:** 10.1371/journal.pone.0142391

**Published:** 2015-11-16

**Authors:** Andrea Vrana, Sabina Hotz-Boendermaker, Philipp Stämpfli, Jürgen Hänggi, Erich Seifritz, B. Kim Humphreys, Michael L. Meier

**Affiliations:** 1 University Hospital of Balgrist, Zurich, Switzerland; 2 Department of Health Sciences and Technology, Human Movement Sciences, ETH Zurich, Switzerland; 3 Department of Psychiatry, Psychotherapy and Psychosomatics, Hospital of Psychiatry, University of Zurich, Zurich, Switzerland; 4 MR-Center of the Psychiatric Hospital and the Department of Child and Adolescent Psychiatry, University of Zurich, Zurich, Switzerland; 5 Division Neuropsychology, Department of Psychology, University of Zurich, Zurich, Switzerland; 6 Center of Dental Medicine, University of Zurich, Zurich, Switzerland; Wadsworth Center, UNITED STATES

## Abstract

Chronic low back pain (chronic LBP) is both debilitating for patients but also a major burden on the health care system. Previous studies reported various maladaptive structural and functional changes among chronic LBP patients on spine- and supraspinal levels including behavioral alterations. However, evidence for cortical reorganization in the sensorimotor system of chronic LBP patients is scarce. Motor Imagery (MI) is suitable for investigating the cortical sensorimotor network as it serves as a proxy for motor execution. Our aim was to investigate differential MI-driven cortical processing in chronic LBP compared to healthy controls (HC) by means of functional magnetic resonance imaging (fMRI). Twenty-nine subjects (15 chronic LBP patients, 14 HC) were included in the current study. MI stimuli consisted of randomly presented video clips showing every-day activities involving different whole-body movements as well as walking on even ground and walking downstairs and upstairs. Guided by the video clips, subjects had to perform MI of these activities, subsequently rating the vividness of their MI performance. Brain activity analysis revealed that chronic LBP patients exhibited significantly reduced activity compared to HC subjects in MI-related brain regions, namely the left supplementary motor area and right superior temporal sulcus. Furthermore, psycho-physiological-interaction analysis yielded significantly enhanced functional connectivity (FC) between various MI-associated brain regions in chronic LBP patients indicating diffuse and non-specific changes in FC. Current results demonstrate initial findings about differences in MI-driven cortical processing in chronic LBP pointing towards reorganization processes in the sensorimotor network.

## Introduction

Low back pain (LBP) is a major health problem with a lifetime prevalence of 85% [1]. While the majority of acute LBP patients recover within weeks, a small minority becomes chronic (pain lasts > 6 months). Chronification is accompanied by psychosocial interferences and causes enormous health care expenditure [2]. A wealth of studies have investigated possible etiologies and consequences of chronic LBP by 1) focusing on ‘end organ dysfunction’, suggesting structural and biomechanical abnormalities at the spinal level, as well as functional impairments [3,4,5,6], 2) describing psychosocial and behavioral variables, such as fear avoidance beliefs that strongly predict the transition from acute to chronic LBP [7,8,9,10,11], for review see Linton [12] and by 3) reporting malfunctional neuroplastic changes on the supraspinal level [13,14,15], for review see Wand et al. [16]. Spinal dysfunction and persistent pain may result in altered sensorimotor integration and may subsequently lead to maladaptive cortical changes in motor control [17]. Indeed, reorganization within primary motor cortex (M1) was revealed by showing a discrete loss of trunk representation [14,18] and reduced anticipatory postural adjustments (APAs) were reported in chronic LBP patients [19]. However, evidence on cortical sensorimotor reorganization in LBP patients investigated by neuroimaging is sparse. This is especially true for functional magnetic resonance imaging (fMRI) measures, as it is challenging to investigate brain activity related to whole-body movements in an MR scanner due to the fact that fMRI data quality is strongly sensitive to subject motion. Therefore, the present investigation used motor imagery (MI) of activities of daily living as a proxy for investigating cortical sensorimotor processing of motor execution (ME). In accordance to Jeannerod’s [20] ‘simulation or resonance theory of action’ hypothesis, MI, action observation and simple understanding of motor actions correspond to a subliminal activation of the sensorimotor system [21,22]. The MI-network comprises the premotor cortex (including the supplemental motor area [SMA]), the superior and inferior parietal lobule (SPL, IPL), the insula, prefrontal regions as well as subcortical structures such as the basal ganglia, and the thalamus [23,24,25,26]. MI has been studied extensively in healthy subjects [25,27,28,29], especially in motor learning and is used to improve performance in sports [30,31]. Moreover, it may play an important role in neuro-rehabilitation [32,33,34], however evidence for cortical functioning of MI in chronic LBP patients is lacking.

Therefore, the aim of the current study was to investigate the functioning of the MI-network in chronic LBP patients using a visually guided MI paradigm by means of fMRI. Due to the paucity of studies related to MI in chronic LBP patients our hypothesis was confined to expecting differential MI-related activity and functional connectivity (FC) in chronic LBP patients compared to healthy controls (HC), indicating potential maladaptive alterations in the sensorimotor network. A better understanding of sensorimotor reorganization processes in chronic LBP patients might help to broaden the basis for a better understanding of motor control impairments and to develop novel approaches for therapeutic MI-guided interventions.

## Materials and Methods

### Participants

Thirty-three subjects participated in this study. Twenty-nine subjects, fifteen chronic LBP patients (mean age 39.7 years; SD 13.5 years; 4 women) and fourteen HC (mean age 33.6 years; SD 12.6 years; 9 women) were included in the final analysis. Four subjects (two HC and two chronic LBP patients) had to be excluded due to excessive head movements (>2.5 mm). Subjects were recruited by online advertisement and word-of-mouth recommendation. Groups were age- and gender-matched (Mann-Whitney U-test for age and Fisher-Yates-test for gender, p>0.05). One patient was left-handed, all others were right-handed according to the Edinburgh Inventory for the assessment and analysis of handedness [35]. The fMRI-images of the left-handed subject were flipped to account for possible differential activations related to handedness. Inclusion criteria for patients were non-specific chronic LBP (neither traumatic nor inflammatory in origin) with a duration of longer than six months. The Pain Detect Questionnaire [36] was used in order to assure that patients fulfilled the inclusion criteria (e.g. no radiculopathy). Exclusion criteria for the HC were acute and/or recurrent back pain within the last six months and past chronic pain episodes. No participants had a history of psychiatric or neurological disorder. All subjects provided written informed consent for the participation in the experiment that was part of a study approved by the Ethics Committee of the Canton of Zurich (KEK-ZH 2012-0029) and conducted in accordance with the Declaration of Helsinki. Subjects were financially compensated for their participation.

### Study design

#### Behavioral assessments

Preceding the fMRI experiment, all participants completed the State and Trait Anxiety Inventory (STAI) which measures state and trait anxiety levels [37]. To assess LBP complaints, patients were asked about location and duration of their back pain and they filled in the Pain-Detect questionnaire [36] which collects data on pain quality and measures pain intensity on an 11-point Numeric Rating Scale (NRS). In addition, patients filled in the Bournemouth questionnaire [38] for the assessment of psychosocial factors and the Tampa Scale of Kinesiophobia (TSK) questionnaire [8].

#### Experimental protocol

**Fig 1:**
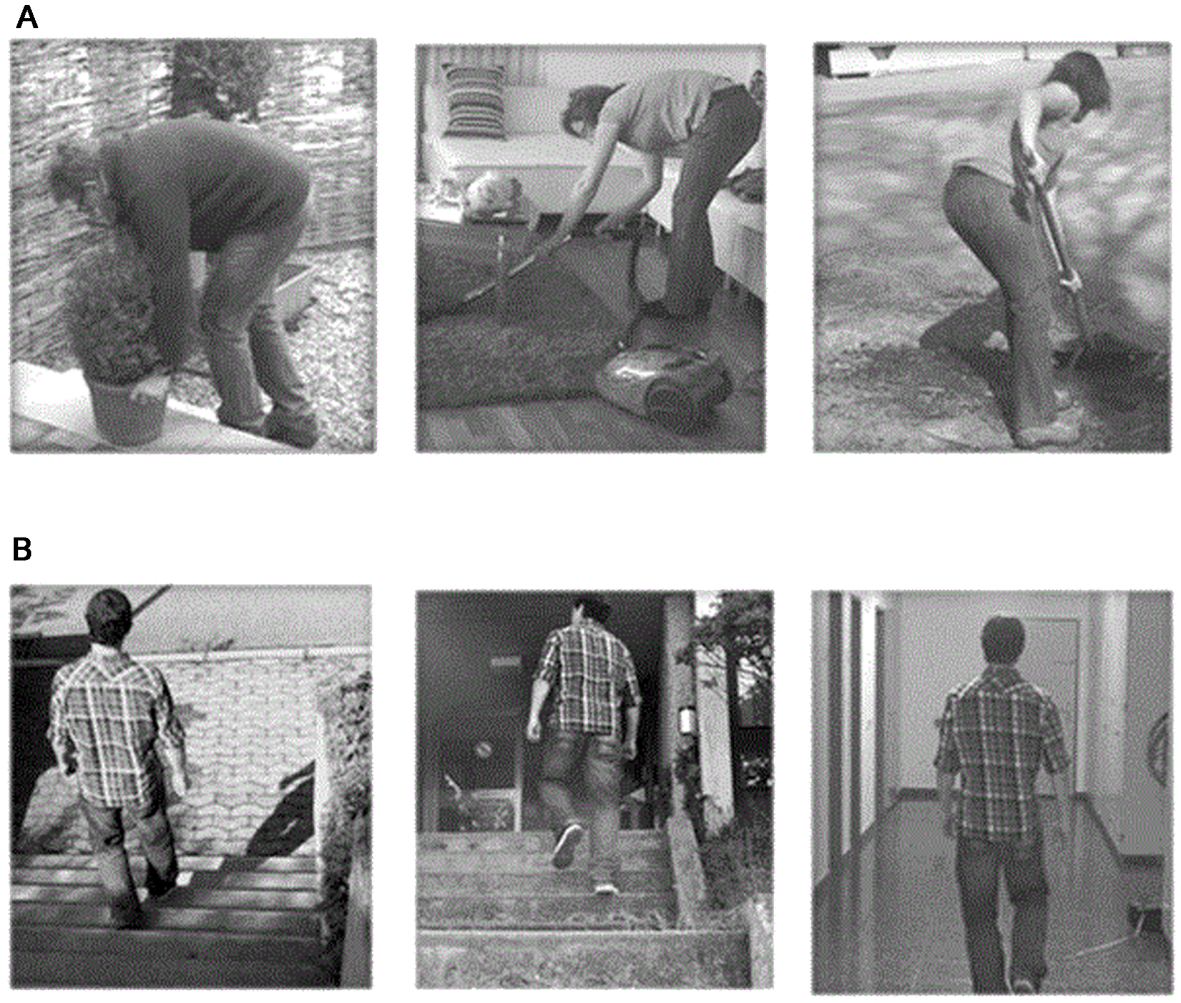
The six presented video clips. A) Activities of daily living (“Activities”) and B) Walking activities (“Walking”).

The stimuli presented during the fMRI measurements consisted of 4s video clips showing whole-body activities of daily living (“Activities”) as well as walking (“Walking”). Activities of daily living were shoveling soil, lifting a flowerpot and vacuum cleaning under a coffee table, whereas the walking videos were comprised of walking on even ground as well as walking up and down on stairs (Fig 1). The video clips were recorded from a third-person perspective which corresponded to the representation of the movement as if the subject was a spectator and another person performed the action [11,39]. The task consisted of visually guided MI as it has been shown that MI under visual guidance (i.e. simultaneous action observation) elicits stronger cortical activations within the ME network than conventional MI [40,41,42,43]. While watching the video clips, both HC and chronic LBP patients were instructed to imagine themselves performing the activities without actually executing them. Preceding the scanning session both groups performed visually guided MI of each activity once. The chronic LBP patients additionally rated the painfulness of those activities on a visual analogue scale (VAS) which was anchored with the endpoints ‘no pain’ (0) and ‘worst pain ever’ (10). During the scanning session video clips were presented by using MR compatible goggles (Resonance Technology Inc., Northridge, USA) that were connected to a notebook computer running the Presentation software (Neurobehavioral Systems, Davis, CA). The fMRI session consisted of 30 trials. Each of the six video clips was presented five times in pseudo-randomized order (no more than two identical consecutive trials). After each video clip, subjects rated the vividness of MI performance of the specific activity on a VAS which was anchored with the endpoints ‘very bad’ (0) and ‘very good’ (10). The ratings were carried out by using an MR compatible trackball (Current Designs Inc., Philadelphia, USA) that moved a cursor on the displayed VAS scale. The rating task was followed by an inter-stimulus interval (ISI: black screen with a fixation cross) of a duration jittered between 6.5 and 8.5 s that was used as baseline.

#### Neuroimaging data acquisition

All measurements were performed on a 3-T whole-body MRI system (Philips Achieva, Best, the Netherlands) equipped with a 32-element receive head coil and MultiTransmit parallel RF transmission. Each imaging session consisted of a survey scan, a B1 calibration scan (for MultiTransmit), a SENSE reference scan and a high-resolution T1-weighted anatomical scan followed by the fMRI acquisition.

fMRI data were acquired with a whole brain gradient-echo echo planar imaging (EPI) sequence (365 dynamic scans) consisting of 37 slices in axial direction with the following parameters: field of view = 240 x 240 mm^2^, matrix = 96 x 96 pixels, slice thickness = 2.8 mm, voxel size 2.5mm x 2.5mm no slice gap, repetition time (TR) = 2100 ms, echo time (TE) = 30 ms, SENSE factor = 2.5, flip angle 80°, interleaved slice acquisition. The number of transversal oriented slices was maximized with respect to the selected repetition time and gradient performance. The resulting field of view in slice direction did not allow an inclusion of the entire cerebellum. Anatomical data were obtained with a 3D T1-weighted turbo field echo scan consisting of 145 slices in sagittal orientation with the following parameters: field of view = 230 x 226 mm^2^, slice thickness = 1.2 mm, acquisition matrix = 208 x 203 pixels, TR = 6.8 ms, TE = 3.1 ms, flip angle = 9°, number of signal averages = 1.

### Statistical neuroimaging analysis

#### Image preprocessing

SPM8 (release v4010, http://www.fil.ion.ucl.ac.uk/spm) software package running on MATLAB R2010b (Mathworks, Natick, MA) was used for fMRI data preprocessing and statistical voxel-by-voxel analysis. EPI volumes of each subject were corrected for differences in head motion, spatially normalized according to the Montreal Neurological Institute (MNI) space and finally smoothed with an 8 mm full-width at half-maximum Gaussian kernel. To control for head movement effects, individual movement parameters (translations in x, y and z-direction, as well as rotations around x, y, and z axis) were implemented in the 1st level model as regressors of no interest. Excessive head motion was defined as a dislocation of more than once in the in-plane voxel resolution (>2.5mm). For removing the low frequency noise a high-pass filter with a cut-off of 128s was used. Trials were modeled as a boxcar response function and convolved with the standard canonical hemodynamic response function (HRF) as implemented in SPM8.

#### Event-related brain activity analysis

For the 1st level analysis the general linear model (GLM) was fitted for each subject by a design matrix composed of the onsets and duration (4 s) of the “Activities” and “Walking” video clips. For each subject, parameter estimates (beta) and contrast images (cons) were computed. The resulting images were analyzed using a random-effects model to allow for population inferences [44]. First, whole-brain activations pooled across conditions (”Activities” and “Walking”) were extracted for each group. Subsequently, between group analyses using two-sample T-tests were computed for the contrasts “Activities > baseline” and “Walking > baseline”. The variance between groups was assumed to be unequal. Error covariance components were estimated using restricted maximum likelihood as implemented in SPM8. To control for false positive results a cluster-based false discovery rate (FDR) correction based on the Gaussian Random Field Theory [45] with a FDR of q <0.05 was used.

#### Event-related functional connectivity analysis

**Fig 2:**
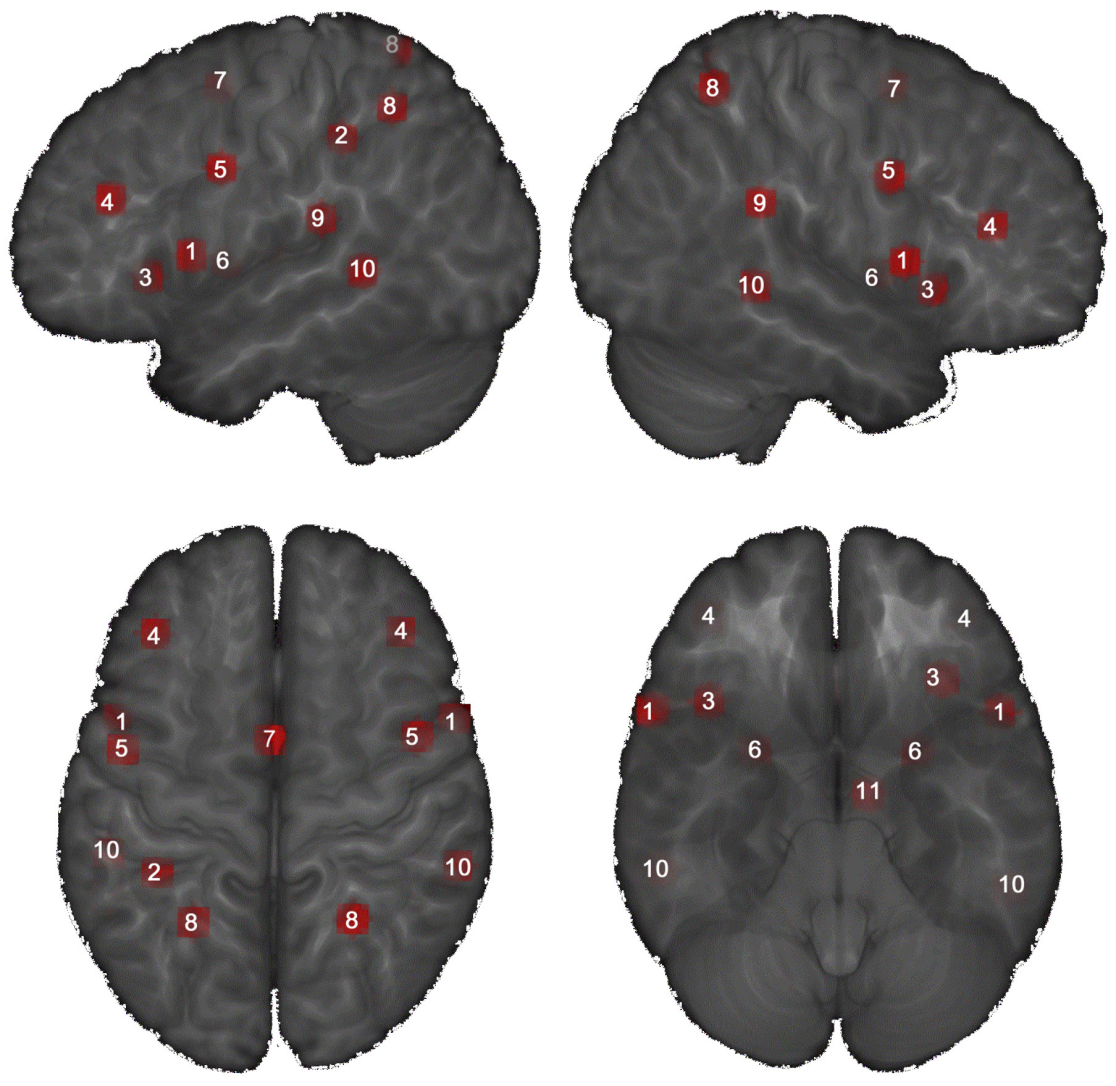
An overview of the motor imagery network based on the meta-analyzes of Hétu and colleagues (2013). MNI-peak coordinates are surrounded by a 6mm-sphere. Views: sagital and axial. 1 Inferior Frontal Gyrus (IFG), bilateral; 2 Inferior Parietal Lobule (IPL), left; 3 Insula, bilateral; 4 Middle Frontal Gyrus (MFG), bilateral; 5 Precentral Gyrus (M1), bilateral; 6 Putamen, bilateral; 7 Supplementary Motor Area (SMA), left; 8 Superior Parietal Lobule (SPL), bilateral; 9 Supramarginal Gyrus (SMG), bilateral; 10 Superior Temporal Sulcus (STS/STG), bilateral; 11 Thalamus, left.

The main advantage of psycho-physiological-interactions (PPI) analysis is that it assesses covariance between regions across time and therefore provides a test of task effects on connectivity. Furthermore, as stated by Gerchen et al., PPI analysis “allows extracting additional information about the brain, which might not be apparent in regional brain activation alone” [46]. The generalized form of the context-dependent PPI approach (gPPI) increases the flexibility of the statistical modelling and improves single-subject model-fit, thereby increasing the sensitivity to true positive findings and a reduction in false positives [47]. For each subject, the deconvolved time course was averaged across 6mm-spheres of MI associated brain regions. Peaks of the MI seed regions were taken from a meta-analysis of MI network activations reported by Hétu and colleagues [23]: left supplementary motor area (SMA), bilateral precentral gyrus (M1), left inferior parietal lobule (IPL), bilateral superior parietal lobule (SPL), bilateral supramarginal gyrus (SMG), bilateral putamen, bilateral middle frontal gyrus (MFG), bilateral inferior frontal gyrus (IFG), bilateral insula and left thalamus (Fig 2). In addition, the superior temporal region (superior temporal gyrus and sulcus [STG/STS]) was also included as a seed region because this brain region is consistently activated in action observation and imitation [48]. Further, to control for possible enhanced overall brain connectivity of the chronic LBP patients, the primary auditory cortex was used as an additional MI-unrelated seed region. Whole-brain functional connectivity analysis was performed using each of the eleven seed regions as well as the additional seed of the primary auditory cortex which was taken from the WFU-pickatlas, Brodmann Area 17, bilaterally. Subsequently, separate psychological terms (”Activities” and “Walking” video clips), physiological regressors (time courses of seed regions) and their PPI interaction terms, as well as the movement parameters, were included in the PPI model. The resulting PPI connectivity estimates were then taken into a two-sample T-test to investigate group differences. Identified clusters were considered to be significant when falling below a cluster-corrected FDR of q < 0.05.

#### Behavioral analyses

SPSS Statistics version 19 software (http://www-01.ibm.com/software/analytics/spss/) was used for the statistical analyzes of the behavioral data. Questionnaires and rating data were verified for normal distribution using the Kolmogorov-Smirnov test. Based on normal distribution, two-sample T-tests were performed for comparisons of the STAI questionnaire between groups. To test for differences in the rating data a repeated measurement ANOVA with within-subject factor “type of movement” and between-subject factor “group” was conducted. Additionally, to exclude interrelationships Spearman correlations were computed between the different questionnaires as well as between ratings. The significance level was set at p<0.05.

## Results

### Behavioral results

#### Questionnaires

Behavioral data was normally distributed. The State and Trait scales did not reveal significant differences between the two groups (State: HC mean=42.4, SD=3.2; chronic LBP mean=44.5, SD=5.3; p>0.202 and T=-1.314; Trait: HC mean=42.8, SD=3.0; chronic LBP mean=43.3, SD=6.9; p>0.807 and T=-0.247). In the chronic LBP group, the Pain Detect Questionnaire showed an overall mean score of 11.2 (SD 6.6) and indicated a mean pain intensity of 4.8 (SD 1.9, range 1.0-7.0). Furthermore, the TSK questionnaire yielded a mean of 37.9 (SD 5.9) and the Bournemouth Questionnaire a mean of 22.1 (SD 11.6).

#### Ratings

Mean ratings of vividness in the HC group were 6.1 (SD 2.5) for the “Activities” and 8.0 (SD 1.6) for the “Walking” videos. The chronic LBP group showed a mean rating of 5.7 (SD 2.4) for the “Activities” and 7.7 (SD 2.1) for the “Walking” videos. The repeated measure ANOVA for the ratings of the MI performance revealed a main effect of condition (“Activities” vs. “Walking”, F=10.153, p<0.005), whereas no interaction effect (type of movement*group) was found (F=0.019, p>0.8). The pain ratings of the chronic LBP group revealed a significantly greater mean of 5.9 (SD 2.5) for the “Activities” compared to a mean of 1.6 (SD 1.7) for the “Walking” videos revealed by a paired T-test (T=7.079, p<0.001).

#### Correlations

Spearman correlations were conducted for the patient-specific questionnaires (Bournemouth, TSK, pain intensity) and the ratings. No significant relationships were found in any of those correlations.

### fMRI results

#### Event-related activity

**Fig 3:**
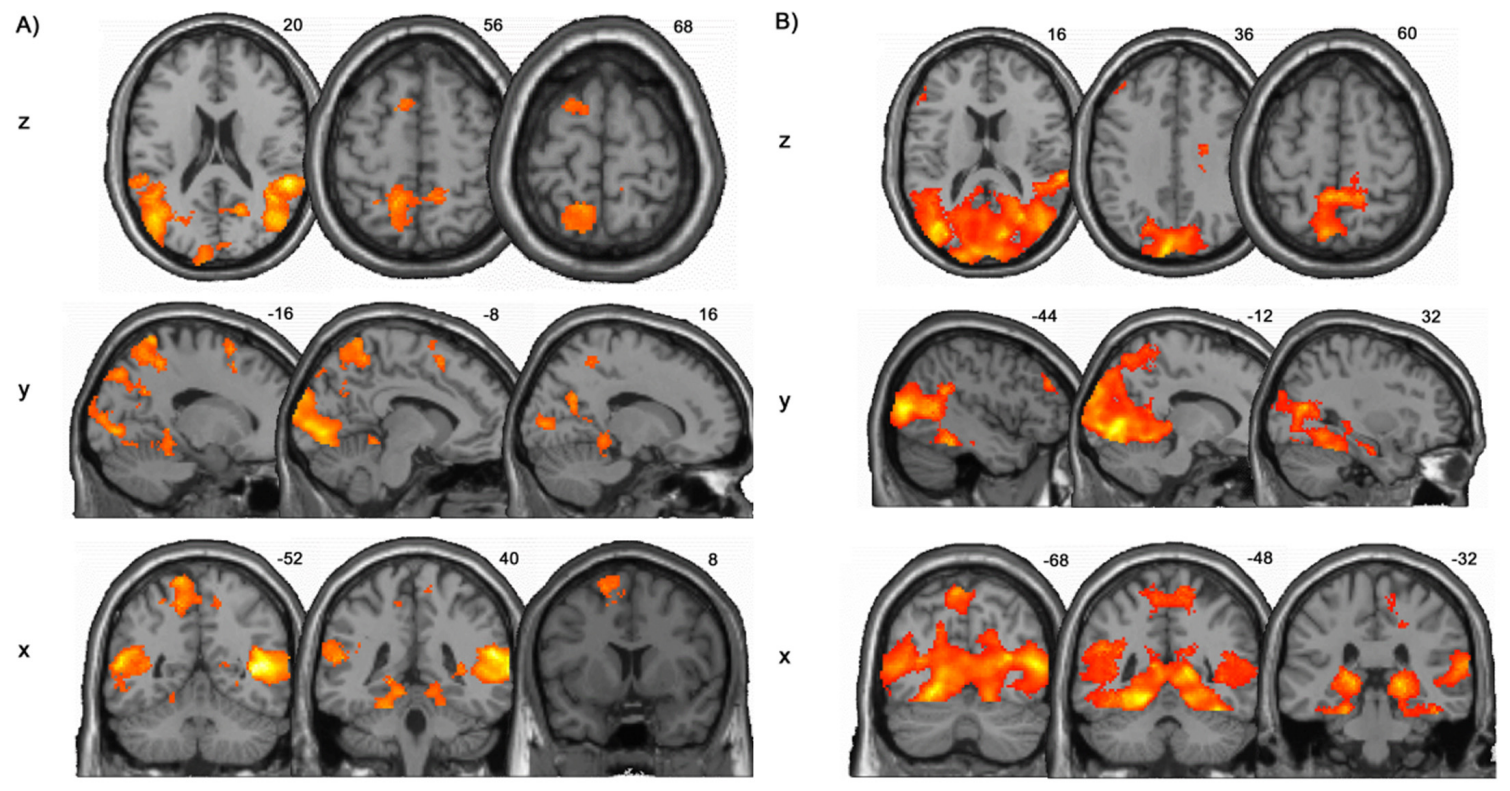
Pooled (“Activities” and “Walking” together) task-related activity per group. A) Healthy controls (HC) and B) chronic Low Back Pain (LBP) patients (q<0.05, false discovery rate corrected, FDR).

The pooled (”Activities” and “Walking”) whole-brain activity in both groups revealed activations in frontal, temporal and parietal cortices as well as in the occipital lobe (FDR-corrected with q <0.05). In the HC group, frontal lobe activations were detected in the middle frontal gyrus, which correspond to the SMA, temporal lobe activations were located in the STG and middle temporal gyrus (MTG), and parietal lobe activations were observed in the IPL and SPL (precuneus). Activations in the occipital lobe were located in the cuneus and lingual gyrus. Subcortically, activity in the thalamus was observed (Table 1, Fig 3A). The chronic LBP group exhibited frontal lobe activation in the dorsolateral prefrontal cortex (dlPFC), the temporal lobe activations were only observed in the MTG and parietal lobe activity was detected in SPL and IPL. Occipital activations were located in the lingual gyrus. Thalamus activity could also be observed (Table 2, Fig 3B).

**Table 1:** Brain Activity of the healthy controls (HC) group, peak activations. Cluster maxima of brain activity of the video clips of activities of daily living (“Activities”) and walking activities (“Walking”) together. All results are false discovery rate corrected (FDR, q<0.05). MNI = Montreal Neurological Institute, STG = superior temporal gyrus, MTG = middle temporal gyrus, MFG = middle frontal gyrus, OL = occipital lobe, SPL = superior parietal lobule, IPL = inferior parietal lobule, PreCG = precentral gyrus, DLPFC = dorsolateral prefrontal cortex.

1	HC Group, “Activities” and “Walking” > baseline, N = 14					
Cluster size	q(FDR)	Z	MNI Peak coordinates			Brain region
5248	<0.001	6.52	60	-44	16	Right STG
		6.07	50	-68	14	Right MTG
		3.63	20	-32	2	Right Thalamus (Pulvinar)
11359	<0.001	6.27	-46	-68	14	Left MTG
		5.51	-10	-80	-2	Left OL (lingual gyrus)
		4.95	-8	-96	26	Left OL (cuneus)
		4.90	-10	-64	62	Left SPL (precuneus)
		4.54	12	-46	58	Right SPL (precuneus)
		4.21	-60	-42	24	Left IPL
		3.51	12	-22	68	Right PreCG
157	<0.01	5.02	42	-42	-18	Right fusiform gyrus
		4.45	36	-22	-14	Right hippocampus
133	<0.02	4.08	-10	12	52	Left MFG (SMA)

**Table 2:** Brain activity of the chronic Low Back Pain (LBP) group, peak activations. Cluster maxima of brain activity of the video clips of activities of daily living (“Activities”) and walking activities (“Walking”) together. All results are false discovery rate corrected (FDR, q<0.05). MNI = Montreal Neurological Institute, STG = superior temporal gyrus, MTG = middle temporal gyrus, MFG = middle frontal gyrus, OL = occipital lobe, SPL = superior parietal lobule, IPL = inferior parietal lobule, PreCG = precentral gyrus, DLPFC = dorsolateral prefrontal cortex.

2	Chronic LBP Group, “Activities” and “Walking” > baseline, N = 15					
Cluster size	q(FDR)	Z	MNI Peak coordinates			Brain region
33236	<0.001	6.96	-14	-82	-4	Left OL (lingual gyrus)
		6.41	-20	-46	-14	Left fusiform gyrus
		6.27	46	-72	14	Right MTG
		6.03	18	-32	0	Right Thalamus
		5.39	22	-44	-16	Right fusiform gyrus
		5.12	34	-12	-22	Right Hippocampus
		5.01	0	-68	58	Left SPL (precuneus)
		4.97	16	-70	30	Right IPL (precuneus)
247	<0.01	4.97	-52	34	20	Left DLPFC

Direct comparison between groups (two-sample T-tests) on the whole-brain level yielded significantly stronger activity in the right STG of the HC group for both contrasts „Activities > baseline“ (q<0.001 FDR-corrected; Z=5.54; MNI peak coordinates 60 -44 16; cluster size 267) and „Walking > baseline“ (q<0.05, FDR-corrected; Z=4.21; MNI peak coordinates 68 -44 12; cluster size 66;). Furthermore, the contrast “Walking > baseline” revealed also enhanced activity in the left SMA (q<0.05, FDR-corrected; Z=3.93 MNI peak coordinates -18 6 70; cluster size 64) in the HC group.

#### Functional connectivity

The majority of seed-regions demonstrated significantly higher FC within the MI network in chronic LBP patients compared to the HC group for the contrasts “Activities > baseline” and “Walking > baseline”, with few exceptions (see Tables 3–6 for details, FDR-corrected with q<0.05). In the HC group compared to the chronic LBP patients only the thalamus revealed enhanced FC with the MTG in the contrast “Activities > baseline”. The control analysis with the auditory cortex as a seed region revealed no significantly enhanced FC of the chronic LBP patients in the “Activities>baseline” condition. The contrast “Walking>baseline” yielded small clusters located in the MFG and STG/STS (n=3; extent threshold in voxel: 105, 85 and 49; FDR-corrected with q<0.05).

**Table 3:** Functional connectivity cluster maxima of the chronic Low Back Pain (LBP) group vs. healthy controls (HC), contrast activities of daily living (“Activities”). All results are false discovery rate corrected (FDR, q<0.05). MNI = Montreal Neurological Institute, STG = superior temporal gyrus, ITG = inferior temporal gyrus, MTG = middle temporal gyrus, MFG = middle frontal gyrus, SFG = superior frontal gyrus, SMG = supramarginal gyrus, OL = occipital lobe, SPL = superior parietal lobule, IPL = inferior parietal lobule, PreCG = precentral gyrus, PCL = paracentral lobule, SMG = supramarginal gyrus.

3	Chronic LBP > HC, “Activities” > baseline, N = 29						
seed	cluster size	q(FDR)	Z	MNI Peak coordinates			Brain region
**IPL**	74	<0.001	4.58	20	-2	64	Right MFG (SMA)
**IFG**	159	<0.001	4.48	-16	-78	54	Left SPL
	75	<0.02	4.17	-10	2	74	Left SFG (SMA)
	210	<0.001	3.92	8	-52	68	Right SPL (precuneus)
**Insula**							No significant results
**M1**	83	<0.007	4.97	58	-58	-16	Right ITG
	92	<0.006	4.07	8	-50	56	Right SPL (precuneus)
	160	<0.001	3.84	32	-70	46	Right SPL
**MFG**	58	<0.04	4.71	32	-76	52	Right SPL
	66	<0.04	4.14	-48	-18	48	Left PCG (S1)
**Putamen**	158	<0.001	4.11	2	-38	66	Right PCL
**SMA**	204	<0.001	4.43	18	-74	26	Right IPL (precuneus)
	155	<0.001	4.34	-16	-72	24	Left IPL (precuneus)
	53	<0.04	4.06	-54	22	34	Left MFG
	47	<0.04	3.93	-10	-4	14	Left Thalamus
	94	<0.003	3.90	-58	-54	20	Left STG
**SPL**	85	<0.007	4.22	-42	-72	36	Left IPL (precuneus)
	73	<0.02	4.04	-26	2	62	Left MFG
	231	<0.001	3.97	-58	-54	18	Left STG
	94	<0.006	3.87	64	-38	-6	Right MTG
	60	<0.03	3.70	22	-72	54	Right SPL
**STG**	210	<0.001	4.02	-10	4	74	Left SFG (SMA)
	54	<0.04	3.88	14	6	74	Right SFG (SMA)
	78	<0.02	3.84	16	-60	60	Right SPL
**SMG**	888	<0.001	4.31	-42	-34	56	Left PCG (S1)
	348	<0.001	4.09	42	-26	44	Right PCG (S1)
	259	<0.001	4.01	-16	-6	68	Left SFG (SMA)
**Thalamus**							No significant results

**Table 4:** Functional connectivity cluster maxima of the chronic Low Back Pain (LBP) group vs. healthy controls (HC), contrast walking activities (“Walking”). All results are false discovery rate corrected (FDR, q<0.05). MNI = Montreal Neurological Institute, STG = superior temporal gyrus, ITG = inferior temporal gyrus, MTG = middle temporal gyrus, MFG = middle frontal gyrus, SFG = superior frontal gyrus, SMG = supramarginal gyrus, OL = occipital lobe, SPL = superior parietal lobule, IPL = inferior parietal lobule, PreCG = precentral gyrus, PCL = paracentral lobule, SMG = supramarginal gyrus

4	Chronic LBP > HC, “Walking” > baseline, N = 29						
seed	cluster size	q(FDR)	Z	MNI Peak coordinates			Brain region
**IPL**							No significant results
**IFG**	159	<0.001	4.48	16	10	62	Right SFG (SMA)
**Insula**	95	<0.02	4.33	14	-48	54	Right SPL (precuneus)
**M1**	124	<0.02	3.84	-6	-2	68	Left SFG (SMA)
**MFG**							No significant results
**Putamen**	124	<0.02	3.95	-62	-28	20	Left pars opercularis (S2)
	56	<0.05	3.55	62	-18	12	Right pars opercularis (S2)
**SMA**	92	<0.006	4.41	-56	-50	20	Left STG
**SPL**	92	<0.006	4.34	-6	-36	72	Left PCL
	58	<0.03	4.21	-66	-38	8	Left STG
	56	<0.03	4.08	-48	-18	48	Left PCG (S1)
	80	<0.008	4.08	68	-24	34	Right SMG (S2)
	140	<0.002	3.95	10	6	70	Right SFG (SMA)
**STG**	103	<0.003	4.33	-62	-14	-6	Left MTG
	83	<0.007	4.09	-10	8	68	Left SFG (SMA)
	174	<0.001	4.03	-12	-28	68	Left PCL
	114	<0.003	3.89	22	-30	70	Right PCG (S1)
**SMG**							No significant results
**Thalamus**	218	<0.001	4.55	14	-34	72	Right PCG (S1)

**Table 5:** Functional connectivity cluster maxima of the healthy controls (HC) vs. chronic Low Back Pain (LBP) group, contrast activities of daily living (“Activities”). All results are false discovery rate corrected (FDR, q<0.05). MNI = Montreal Neurological Institute

5	HC > chronic LBP, “Activities” > baseline, N = 29						
seed	cluster size	q(FDR)	Z	MNI Peak coordinates			Brain region
**Thalamus**	65	<0.04	3.94	42	-66	12	Right OL (right MTG)
**All other**							No significant results

**Table 6:** Functional connectivity cluster maxima of the healthy controls (HC) vs. chronic Low Back Pain (LBP) group, contrast walking activities (“Walking”). All results are false discovery rate corrected (FDR, q<0.05). MNI = Montreal Neurological Institute

6	HC > chronic LBP, “Walking” > baseline, N = 29						
seed	cluster size	q(FDR)	Z	MNI Peak coordinates			Brain region
**All**							No significant results

## Discussion

The present fMRI-study aimed at investigating the functioning of the MI network in chronic LBP patients. Visually-guided MI of daily activities was used in order to disentangle expected differential neural sensorimotor processing among HC and chronic LBP patients. As a novel finding the MI-driven activity yielded reduced brain activation within the SMA and STG/STS, while the connectivity analysis indicated significantly enhanced FC within the MI-network in chronic LBP compared to the HC group.

### Event-related activity

The simulation theory of Jeannerod [20] postulates that MI involves similar brain areas as ME and even sole action observation. Based on this assumption MI was used as a proxy for ME of daily activities in order to investigate the functioning of sensorimotor networks in chronic LBP patients. The results of the event related activity analysis of both groups are in keeping with known MI associated brain activity involving a fronto-parietal network and subcortical structures such as the thalamus [23,24,48]. However, M1 activity could not be demonstrated, supporting the rather ambiguous role of M1 in MI [23]. Due to the visual-guidance of the MI task, extensive activations were also observed in visual and visuo-motor areas in both groups.

Regarding the behavioral measures, both groups rated the vividness of MI performance equally indicating that chronic LBP patients retained their ability to perform mental movements. In contrast, on the neural level the hypothesis of maladaptive alterations in the sensorimotor processing was confirmed by demonstrating significant differences in the functioning of the MI-network in chronic LBP patients regarding brain activity as well as connectivity patterns. Importantly, pain ratings of the chronic LBP group indicated that patients experienced only the MI-performance of the “Activities” as painful [49] while the MI of the “Walking” condition was not painful.

With respect to the brain activations the between group (two-sample T-tests) comparisons on the whole-brain level revealed reduced activity within two brain regions in chronic LBP patients, namely the left SMA and right STG/STS.

While robust MI-driven SMA activity was observed in HC subjects, it was completely absent in chronic LBP patients even at an uncorrected threshold of p<0.001. The SMA represents an inherent part of the MI-network [23,24,48] and is linked to adequate motor planning and voluntary motor control [50,51,52], for review see [53]. With respect to the stimuli i.e. whole-body activities as presented in the current study, there is also considerable evidence for the involvement of SMA in postural control especially in anticipatory postural adjustments (APAs) and related body balance control [54,55]. In particular, SMA was activated when pressure stimuli were applied to the lumbar spine indicating fine scaled motor preparation/trunk stabilization mechanisms in the absence of an intended or actual performance, verifying the crucial role of this region in anticipatory postural control [56]. In chronic LBP patients, Jacobs and colleagues revealed delayed APAs suggesting a decrease in postural control and stability [19]. Furthermore, significantly improved APAs resulted from the application of repetitive transcranial magnetic stimulations (rTMS) over the SMA in a neurologic patient group with considerable APA delays [55]. Together, these findings suggest a direct involvement of the SMA in trunk movement coordination. Therefore, the demonstrated maladaptive functioning of the SMA revealed by MI might be based on progressive dysfunction of motor circuits and thus provides a mechanism to explain reported impairments in postural control in chronic LBP patients.

However, it remains to be explored to which extent clinically relevant impairments of postural control in chronic LBP patients [57] might be linked with maladaptive brain mechanisms.

Extensive STG/STS activation was observed as a consequence of the visual guidance (e.g. action observation) of the MI-task. Interestingly, when contrasted against baseline STG/STS activity during “Activities” and “Walking” was significantly reduced in chronic LBP patients compared to HC. The STG/STS responds to images of human bodies and their orientations [58,59] and is known to play an important role in the understanding and interpreting of human movements as well as in matching sensory inputs with internal movement representations [60,61,62,63]. Correspondingly, MI and ME require a high amount of sensory input processing in order to provide a real-time representation of the body (e.g. body-scheme) [64,65]. In order to perform MI and ME appropriately, the matching of the internal representation of a person’s body with an intended movement is realized by comparing the predicted sensorimotor consequences of the action and the actual sensorimotor feedback [66]. In chronic LBP patients, altered sensory input characterized e.g. by reduced two-point discrimination and altered body-scheme has been previously reported [67,68,69,70,71]. Furthermore, it has been revealed that chronic LBP patients showed a reduced ability to discriminate weights in possibly hurtful trunk movements (e.g. lifting objects) in human point-light biological motion displays [72,73]. As their general ability in visual discrimination tasks regarding biological motion in point-light displays remained unharmed [73], their constraints seemed to be limited only to trunk movements. The impairment of sensory discrimination and subsequently restrained interpretation of trunk-related visual movement tasks might be linked with reduced STG/STS activation in the present visually-guided MI task.

### Event-related functional connectivity (FC)

Activity and connectivity analyses are the two main applications of fMRI. As both analyses are based on the measure of changes in hemodynamic responses one could expect a certain overlap of brain patterns for the same task. However, FC changes can occur even if a particular brain area does not appear to be activated throughout a task and vice versa providing additional information about neural functioning [74,75]. In the current investigation we used the gPPI method to reveal FC changes [47] which yielded major differences between the chronic LBP and HC group in the MI-network. Overall, the chronic LBP patients exhibited significantly enhanced MI-driven FC compared to the HC group throughout the MI network. To further control for possibly enhanced whole-brain connectivity within the chronic LBP group we performed an additional control analysis using the primary auditory cortex as a MI-unrelated seed region. The analysis revealed no enhanced FC of the chronic LBP group in the “Activities>baseline” condition. This observation confirms largely MI-related changes in FC whereas an overall enhanced brain connectivity of chronic LBP patients is less probable. The enhanced FC of chronic LBP patients in the MI network can be interpreted in different ways and these explanations are not mutually exclusive. Enhanced FC in the default mode network as well as in the executive attention network might be associated with hyper-excitability as previously demonstrated in fibromyalgia patients, a pathology associated with ongoing diffuse chronic pain state [76]. In addition, the FC strength among these networks correlated with spontaneous pain ratings. With respect to chronic LBP, diffuse and non-specifically enhanced FC across the MI network during a pain free MI-task might indicate a common neural maladaptive process. There is evidence about lower pain thresholds and enhanced functional activity in particular regions involved in pain processing in chronic pain states as well as during acute noxious stimulations [15,18,77]. Furthermore, dysfunctional central pain modulation and central sensitization characterized by e.g. hyper-excitability have been also reported in chronic LBP patients [78,79] and may also have an impact on FC between different brain regions.

Supporting this, studies using electroencephalography (EEG) have shown that the sensorimotor cortical activation, as reflected by EEG alpha rhythms, is modulated during the expectancy of somatosensory stimuli and that patients might exaggerate the anticipatory activation of sensorimotor cortex to negligible noxious stimuli [80,81,82]. Thus, chronic pain might be maintained through hypervigilance toward noxious stimuli due to abnormal attentional cortical-thalamic systems [83].

An alternative explanation for the observed enhanced FC in chronic LBP patients might be based on the high neural demand of a MI task. It has been shown that healthy subjects accommodated to a more demanding task by enhancing FC within the MI-network [28]. Taking into account the non-significant group differences of the ratings about the vividness of the MI-performance, this would imply that chronic LBP patients retained the ability to perform complex MI and ME tasks, however they required more cortical recruitment depicted by enhanced FC in order to perform a task.

### Limitations

Regarding shortfalls of the present study there are mainly methodological issues to mention. We considered the ISI as a baseline although there is no inherent baseline associated with the blood-oxygen-level-dependent (BOLD) signal [84]. We assumed that the baseline represented something akin to a zero-activity condition which was then compared with the activity during the MI-tasks. Next, the canonical hemodynamic response function that was used in the deconvolution step of the gPPI was assumed to be constant across voxels and subjects [85]. Until now, the choice of model for the deconvolution has not been investigated, despite different hemodynamic response functions being used in activation studies [47,86]. Thus, future studies should investigate the effect of variable hemodynamic response functions on PPI, especially focusing on differential hemodynamic responses in patient populations due to possible changes in neurovascular coupling. Furthermore, current fMRI group level statistics are based on an averaged anatomical template. As there is a high degree of variability in inter-subject anatomy and functional localization, the use of spatial alignment and smoothing kernels increases the overlap among subjects but makes it difficult for identifying distinct activity in small brain regions. To reduce inter-subject anatomical variation e.g. in the SMA, specific registration techniques such as DARTEL or HAMMER are recommended [87]. Further, visual MI requires self-visualization of a movement from a first- (internal) or third-person (external) perspective [39]. As the subjects were instructed to imagine themselves performing the activities both perspectives end up in a self-visualization of the subject. However, the observation of a “third person” might have additionally influenced brain activity but this was assumed to be equal in both groups. Finally, the current findings have to be carefully interpreted. To our knowledge this study presents initial findings regarding neural correlates of MI in chronic LBP patients. However, a causal relationship between the current findings of alterations in MI-related brain mechanisms and the disease pattern of chronic LBP patients cannot be demonstrated and remains therefore further investigation.

## Conclusion

The current investigation provides first evidence for obvious differences between chronic LBP patients and HC subjects regarding MI-driven activity and FC. First, reduced activity in the SMA suggests dysfunctional mechanisms regarding motor planning, feed-forward monitoring and related APAs, whereas reduced STG/STS activity indicates deficits in the integration of sensory inputs. Second, the non-specifically enhanced FC within the MI network of chronic LBP patients might indicate pain-driven maladaptive alterations in the sensorimotor network in terms of hyperexcitability or an enlarged need for neural resources. These findings may broaden the basis for the understanding of sensorimotor reorganization processes in chronic LBP patients and might ultimately help developing novel approaches for therapeutic MI-guided interventions.
